# Exercise and large airway issues

**DOI:** 10.14814/phy2.70454

**Published:** 2025-07-10

**Authors:** Zander J. Williams, Giovanni Cenerini, James H. Hull

**Affiliations:** ^1^ Department of Respiratory Medicine Royal Brompton Hospital London UK; ^2^ Department of Surgical, Medical and Molecular Pathology and Critical Care Medicine University of Pisa Pisa Italy; ^3^ Institute of Sport, Exercise and Health (ISEH), Division of Surgery and Interventional Science University College London London UK

**Keywords:** excessive dynamic airway collapse, exercise, large airway, tracheobronchomalacia

## Abstract

The large airways, extending from the trachea to the main bronchi, respond dynamically to exercise‐induced ventilatory demands. Large airway collapse (LAC) represents a spectrum of conditions characterized by excessive reduction in tracheal and/or main bronchial lumen during expiration. Utilizing the most common diagnostic criteria, defined as ≥50% reduction in airway cross‐sectional area during expiration, LAC is a common finding in around one in three patients with underlying lung disease. However, it is also apparent that healthy, asymptomatic people meet this diagnostic criteria. Despite being recognized as a cause of exertional symptoms, the relationship between LAC and exercise‐related symptoms or limitation is currently poorly understood. Traditional clinical approaches use forced expiratory measurements performed at rest during bronchoscopy or imaging studies to assess the condition. But novel tests, visualizing the large airways during exercise, may provide more physiologically relevant insight and are an important next step towards the development of targeted interventions for this clinical entity. This review aims to examine large airway behavior during different ventilatory challenges, with particular focus on comparing exercise hyperpnea with forced expiratory maneuvers.

## 1 INTRODUCTION

During exercise, the respiratory system is challenged to respond to an increased ventilatory demand, dictated by a heightened requirement for oxygen delivery. The airway tract acts as the natural conduit for ventilation and is exposed to mechanical, thermal, and chemical stress of exercise hyperpnea. While several decades of research have evaluated the stress placed on the respiratory system during exercise, much of the work in this area describes the lower airway response to exercise, with a focus on pathophysiological sequelae such as exercise‐induced bronchoconstriction (Aggarwal et al., 2018). More recently, however, there has been increased focus on the potential role of the large airways and the function of this part of the respiratory system during exercise.

The large airways are defined as the segment of the airway tract that extends from the trachea to the main bronchi and bronchus intermedius. These airway segments are primarily considered to function as a conduit facilitating the bulk flow of air to the more distal airway segments. However, this segment of the airway does have a highly evolved structure (i.e., with a cartilaginous ring and posterior trachealis muscle) to permit inward movement of the posterior wall to facilitate airway clearance (Murgu & Colt, 2013).

In some people, it is now evident that inward movement of the large airway may be considered “excessive.” This “so called” large airway collapse (LAC) is typically diagnosed with the criterion of reduction of central airway lumen cross‐sectional area of 50% or greater during expiration (Murgu & Colt, 2013). The presence of LAC appears to be a relatively common finding in imaging studies of people with chronic lung disease (approximately 30%), but importantly, up to 70%–80% of healthy, asymptomatic individuals have been found to meet this diagnostic criteria (Mitropoulos et al., 2021; Boiselle et al., 2009). It is associated with symptoms including shortness of breath, cough, wheeze, and difficulty clearing secretions. However, it is not yet clear if LAC directly explains or contributes to the development of exertional dyspnea and cough in people with lung disease.

This review aims to provide an overview of the large airway response to ventilatory maneuvers, with a specific focus on comparing the response to exercise with other high airway stress maneuvers such as forced expiration. We also explore the relationship between LAC, exercise intolerance, and symptoms.

## 2 TYPES OF LARGE AIRWAY DISORDERS AND PREVALENCE

### 2.1 Definitions and etiology

**FIGURE 1:**
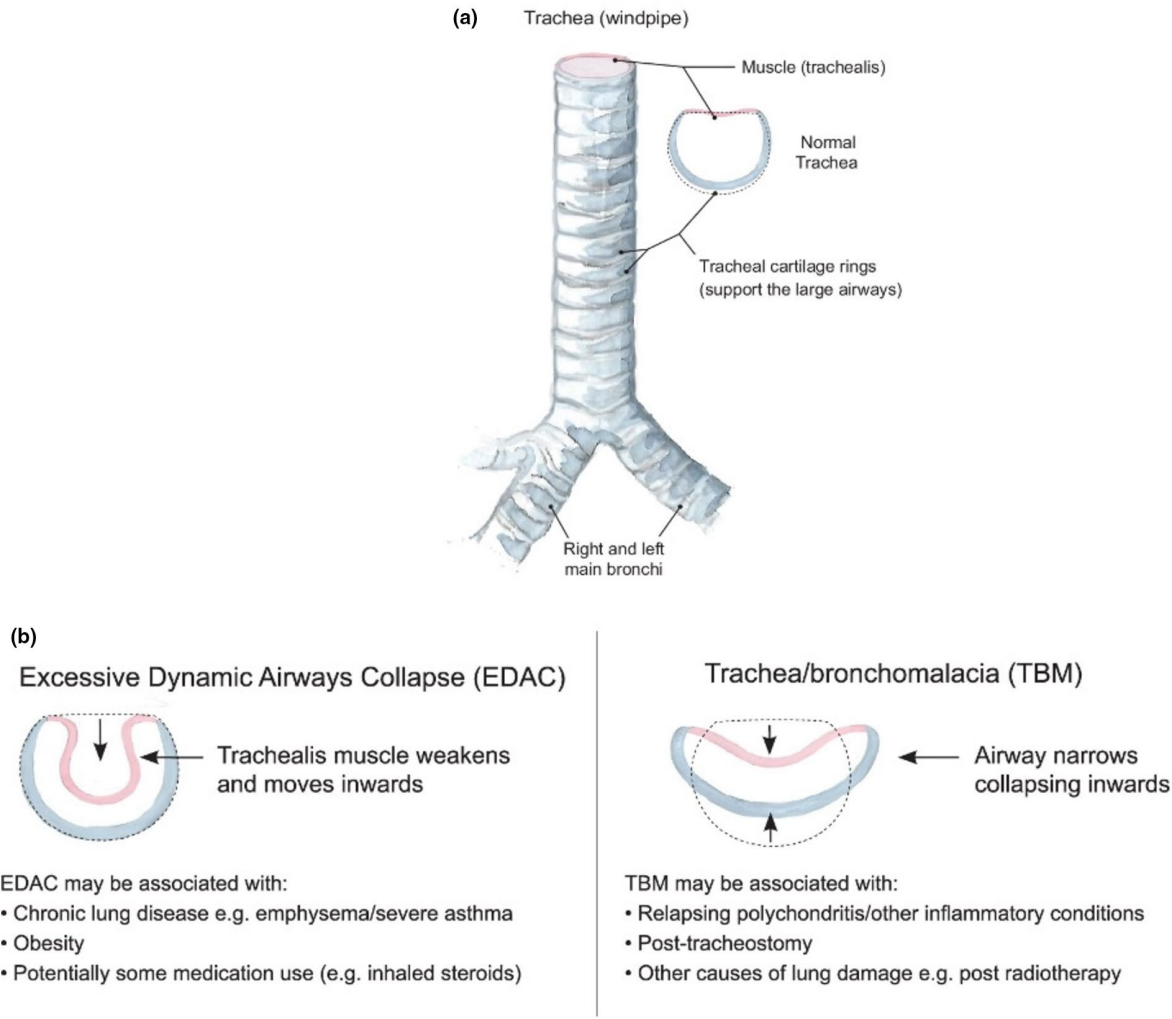
(a) Trachea and main bronchi. (b) Classification of large airway collapse by cross‐sectional airway morphology, with some associated causes. Reprinted with permission of the American Thoracic Society. Copyright © 2025 American Thoracic Society. All rights reserved (Housley et al., 2024).

This term LAC encompasses a spectrum of pathological conditions affecting the tracheobronchial tree, with Tracheobronchomalacia (TBM) and Excessive Dynamic Airway Collapse (EDAC) the most commonly described. TBM is characterized by weakness of the tracheobronchial cartilaginous structures (the rigid support system of the airways), resulting in anterolateral collapse during expiration. Whereas the term EDAC is typically used to describe excessive inward invagination or bulging of the posterior tracheal membrane (the soft tissue wall at the back of the airway) into the airway lumen during expiration, occurring without cartilaginous involvement (Figure 1) (Murgu & Colt, 2013).

The pathophysiology of these conditions involves complex interactions between airway wall properties and ventilatory mechanics. In TBM, histopathological studies have demonstrated structural alterations, including a reduction in the cartilage‐to‐soft tissue ratio from the normal 4.5:1 to as low as 2:1 (Ridge et al., 2011). Additionally, chronic inflammation can lead to cartilage destruction and replacement with fibrous tissue, further compromising airway stability (Jokinen et al., 1977). These structural changes manifest in distinct morphological patterns visible during airway assessment. The most common presentation, known as the crescent or membranous type, presents as anterior displacement of the posterior membrane. Less frequently, the saber‐sheath type presents with lateral wall collapse, while the circumferential type involves collapse of both anterior and lateral walls (Boiselle & Ernst, 2006; Carden et al., 2005).

The lunate tracheal shape, particularly evident during inspiration or the frown‐shaped evident on expiration in patients with TBM, represents a key diagnostic feature identified through imaging studies (Lomasney et al., 1989). The pathophysiology of EDAC centers on altered posterior membrane dynamics during expiration (i.e., crescent shape morphology) and this entity is often encountered in chronic pulmonary disease where there is deemed to be a loss of the scaffolding structures in the lung (e.g., emphysema). Other studies have found that a tendency to LAC may be associated with a raised Body Mass Index (BMI), and this may relate to altered airway wall structure, and/or compliance (Bhatawadekar et al., 2021).

### 2.2 Epidemiology and clinical significance

The true prevalence of LAC has been challenging to establish due to the varying diagnostic criteria used and methodological variations across studies. In this respect, a systematic review by Mitropoulos et al., (2021) highlighted heterogeneity in assessment methods, with imaging modalities being utilized in 80% of studies, despite evaluation during flexible bronchoscopy being considered the gold standard for diagnosis. Most studies and prevalence rates reported have assessed LAC using forced expiratory or coughing maneuvers at rest, with airway narrowing of 50% or more considered diagnostic (Mitropoulos et al., 2021).

Some early bronchoscopic studies identified acquired TBM in around 5% of patients undergoing evaluation (Jokinen et al., 1977). This finding was later expanded through a comprehensive analysis of 4283 patients undergoing bronchoscopy, which documented TBM in around 13% of cases, with severe presentations occurring in 3%. It is also noted that prevalence rates are impacted by aging cohorts, with severe TBM found to increase to 6% in patients over 80 years (Ikeda et al., 1992).

A few studies have used computed tomography (CT) imaging to assess the degree of expiratory tracheal collapse in healthy individuals at rest. One of the earliest studies found LAC was present in up to 78% of healthy individuals during forced expiration (Boiselle et al., 2009). O'Donnell et al., (2012) found mean tracheal collapse of nearly 60% in healthy individuals, with a positive association between the degree of collapse and those that were older/ male. Litmanovich et al., (2010) observed regional variations in the degree of collapse between airway segments, with mean collapse of 67% in the right main bronchus and 61% in the left main bronchus during forced expiration in healthy volunteers, with 73% of participants exceeding the 50% threshold for defining bronchomalacia. In contrast, some studies found that the degree of expiratory collapse is within normal physiological limits (i.e., < 50%) (Dal Negro et al., 2013; McDermott et al., 2009).

A wide range of prevalence of LAC has been reported in patients with underlying lung disease, with collapse >50% found in approximately one third (Mitropoulos et al., 2021). When specific populations are assessed, LAC prevalence varies by condition and patient characteristics. Bronchoscopic studies identified LAC in 53% of patients with chronic bronchitis (Jokinen et al., 1977), while imaging‐based assessments found LAC in 13% of asthma patients (Dal Negro et al., 2013). LAC prevalence increases in patients with multiple risk factors, with dynamic CT studies showing rates of 53% in morbidly obese COPD patients compared to 20% in non‐obese counterparts (Boiselle et al., 2013).

## 3 WHAT IS THE LARGE AIRWAY RESPONSE TO VENTILATORY MANEUVERS?

Much of the work evaluating large airway function has studied the response to forced maneuvers. Physiologically, the behavior of the large airways is dictated by the complex interplay between airway wall properties, transmural pressure gradients, and airflow dynamics. During ventilatory challenges, any tendency to airway collapse is explained by the relationship between intraluminal pressure, pleural pressure, and the inherent structural integrity of the airway walls.

### 3.1 Large airway behavior during forced expiration: Theoretical models

Two complementary theories have been proposed that explain airway collapse during forced expiration.

The Equal Pressure Point (EPP) theory, described firstly by Mead et al., (1967), models how during expiration, airway pressure decreases due to resistance to flow until it equals pleural pressure at a specific point (i.e., EPP). In the context of LAC and during forced expiration, the EPP migrates away from the periphery, creating a progressively longer compressed proximal segment, which increases resistance and limits expiratory flow. The point where pleural pressure exceeds airway pressure, airway compression occurs (i.e., central airway segments). The location of this EPP is influenced by lung elastic recoil, airway resistance, and airway compliance (Murgu & Colt, 2013).

Complementing this explanation, wave‐speed theory describes how pressure waves propagate through the airways like waves moving through water. Maximum airflow is achieved when flow velocity equals the wave‐speed of pressure propagation through the airway walls. The choke point described in wave‐speed theory is equivalent to the juncture of upstream and flow‐limiting segments in EPP theory (Murgu & Colt, 2013).

During forced expiratory maneuvers, the sudden expulsion of air after maximal inspiration generates extreme pressure gradients, with expiratory pressures reaching approximately 145 cmH_2_O (Thomas et al., 1997). This creates substantial stress on the airway walls, especially at points of structural vulnerability such as the posterior membrane of the trachea and main bronchi.

### 3.2 Large airway behavior during exercise

In contrast to the forces exerted during a single forced expiratory maneuver, the ventilatory stress that develops during exercise typically evolves more progressively over several seconds to minutes. At the onset of exercise, there is typically an increase in tidal volume achieved through alterations in end‐expiratory and end‐inspiratory lung volumes, followed by a later increase in breathing frequency, commensurate with higher intensities of exercise (Tipton et al., 2017). The pressure dynamics during exercise ventilation are markedly lower than those during forced expiration, with maximal exercise expiratory pressures reaching approximately 42 cmH_2_O (Thomas et al., 1997).

There are very few studies visualizing the large airway during exercise. Williams et al., (2024) found differences between the large airway response to resting forced expiratory maneuvers compared to exercise. Of 28 healthy volunteers, with normal BMI, who underwent continuous bronchoscopy during exercise (CBE), excessive dynamic airway collapse (tracheal collapse ≥50%) was identified in 16 subjects (64%) on MRI, and in six (24%) individuals during resting bronchoscopy. A finding not evident during CBE. It appears that the healthy airways maintain patency during strenuous physical activity despite marked collapse during forced expiratory maneuvers (Williams et al., 2024). A key conclusion from this paper was suggested that forced expiratory maneuvers may not accurately reflect airway function during physical activity. However, the precise explanation for the differences between these testing methodologies is not yet clear, and more work is needed. It is conceivable that the placement of the bronchoscope acts to splint the airway and provide some form of positive end‐expiratory pressure effect, which may prevent some degree of expiratory collapse. A similar finding was postulated in patients with COPD who underwent laryngoscopy during exercise, where movement of the upper airway aperture was suggested as a mechanism to preserve optimal expiratory flow and to offset PEEP (Baz et al., 2015).

The relationship between LAC and exercise limitation remains incompletely understood, with some patients demonstrating marked symptoms during exercise while others with similar degrees of collapse during forced maneuvers remain asymptomatic (Boiselle et al., 2012). This suggests that the degree of collapse alone may not determine symptomatology, and other factors such as flow dynamics, neural control mechanisms, or compensatory responses may play important roles in determining the functional impact of airway collapse during physical activity.

## 4 CLINICAL MANIFESTATIONS AND IMPACT ON FUNCTIONAL CAPACITY

The presence of LAC is increasingly recognized to underpin respiratory symptoms including exertional dyspnea, cough, wheeze, and difficulty clearing secretions. Given the report of these non‐specific symptoms, LAC may often mimic asthma or COPD and contribute to treatment refractory disease states. However, it is also apparent that in patients with LAC and a respiratory comorbidity, higher rates of symptom severity have been reported. In one large study of over 8000 participants, individuals with LAC reported greater modified medical research council (mMRC) dyspnea scores and worse St George's Respiratory Questionnaire (SGRQ) scores compared to non‐LAC controls (Bhatt et al., 2016), in 241 smokers with and without COPD, LAC was associated with increased mMRC scores independent of CT‐emphysema severity, FEV_1_, and wall area percent of segmental airways (Tanabe et al., 2021). However, contrasting results have been reported in smaller studies, failing to establish a clear link between LAC and dyspnea scores (Leong et al., 2017).

Patients with LAC often present with symptoms such as expiratory wheeze or dyspnea at rest; however, it appears that in some groups, these clinical features can be most prominent on exertion. In this respect, Weinstein et al., (2016) reported findings from six military personnel with no underlying lung disease who reported exertional symptoms. The patients underwent a comprehensive assessment including PFTs, cardiopulmonary exercise testing (CPET), CT imaging, and bronchoscopy. Standard PFTs and CPET failed to identify abnormalities, but CT and exercise bronchoscopy performed on a cycle ergometer revealed exercise‐associated EDAC.

There are very few studies that have assessed the relationship between exercise (in)tolerance and LAC. Boiselle et al., (2012) evaluated COPD patients and found the degree of LAC was not correlated with 6‐min walk test (6MWT) distance, or other markers of physiological function including FEV_1_ percent predicted and transfer factor of carbon monoxide. Represas‐Represas et al., (2015) similarly found no correlation between the degree of collapse and 6MWT distance or MRC dyspnea scale.

The causes of the physiological disconnect between anatomical findings and functional outcomes remain to be determined. Further work evaluating these relationships is required; however, it is likely the results are currently confounded by the use of static assessments that rely on forced expiratory maneuvers that create conditions dissimilar to exercise hyperpnea. Large airway function during forced maneuvers compared to exercise ventilation is different in healthy participants, but we are not yet clear about the precise mechanisms for this finding. Factors such as high pulmonary pressures and mechanical stress during forced expiration compared to exercise are likely causes, but further work is needed. In one of the only studies to evaluate pressure and flow mechanics in a group of patients with TBM, Loring et al., (2007) found no association between collapse severity and expiratory flow limitation. This finding further confounds the data that excessive collapse may cause physiological impairment.

Although it remains difficult to determine whether functional impairment results from LAC or underlying lung disease. It can be assumed that increased resistive load during quiet respiration will intensify with exercise ventilation. Further study is needed to evaluate large airway dysfunction's impact on exercise tolerance, recognizing that breathing pattern disorders and deconditioning likely contribute to symptoms associated with LAC.

## 5 ASSESSMENT APPROACHES

Overall, the approach to diagnosis of LAC requires a systematic evaluation given the non‐specific nature of respiratory symptoms. Although performing forced expiratory maneuvers during flexible bronchoscopy is considered the gold standard test to assess the condition, it appears imaging modalities are more commonly used, and both methods are often used interchangeably. Despite this, a problem remains the lack of consensus regarding the optimal approach and criteria used to diagnose LAC.

### 5.1 Resting assessment

Pulmonary function tests may be performed as part of an initial work‐up for patients being investigated for LAC but are typically non‐diagnostic. In one study of patients with confirmed moderate to severe TBM, 44% presented with airflow obstruction, 18% had a restrictive pattern, and 17% had a mixed defect, while approximately 20% showed normal spirometry. Flow volume loops may provide some additional insight, with the most common findings being low peak expiratory flow (48%), biphasic morphology (20%), expiratory notching (9%) and oscillations (3%) (Majid et al., 2013).

Bronchoscopic visualization permits direct assessment of morphology and severity of collapse during forced expiratory maneuvers and coughing. The reduction in airway lumen cross‐sectional area is estimated by the bronchoscopist, with a collapse diagnostic threshold of 50%. Although operator variability may occur, one series reported favorable inter‐ and intra‐observer agreement (Lee et al., 2007). Despite being considered the gold standard, bronchoscopy is increasingly complemented by imaging modalities that allow non‐invasive assessment.

**FIGURE 2:**
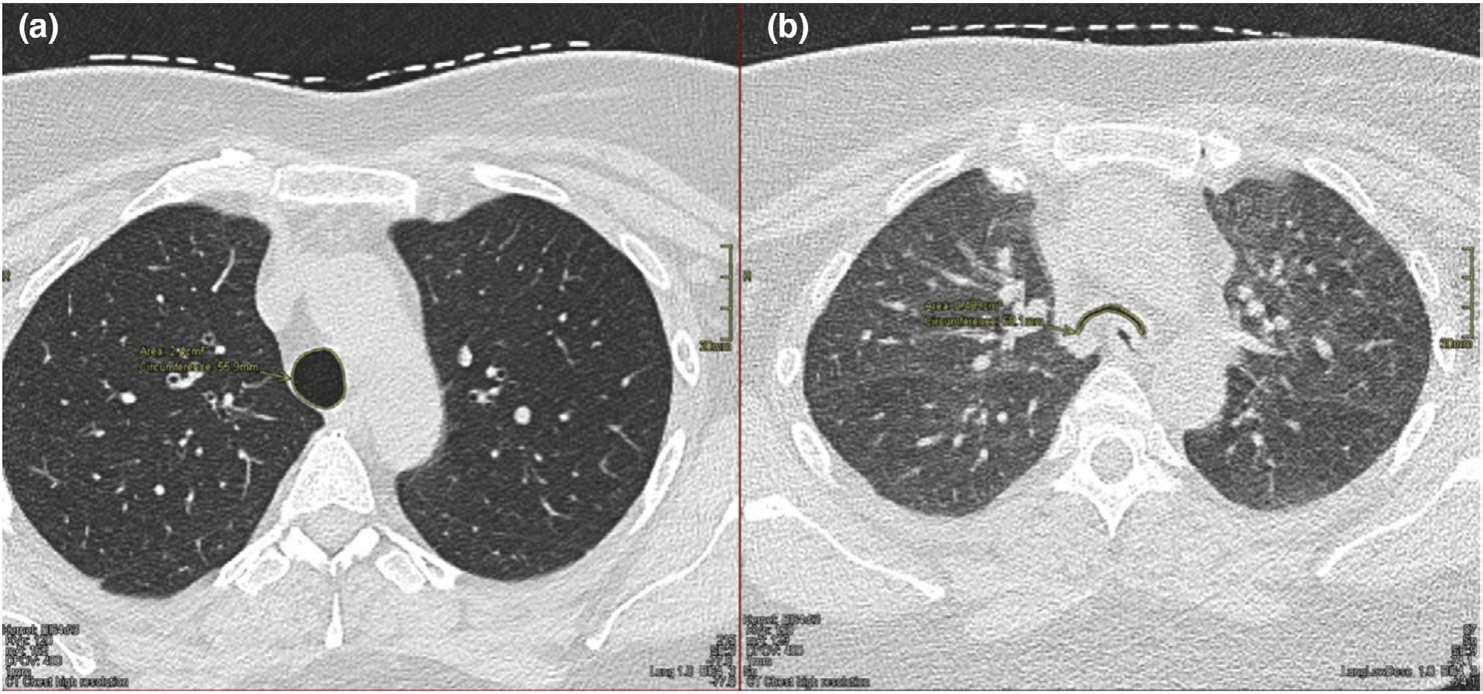
Axial CT imaging of a 53‐year‐old female. (a) End inspiratory CT image of the trachea displaying a round, circular shape, with a cross‐sectional area of 2.8 cm^2^. (b) End expiratory image at a similar tracheal level displaying a bowing, crescent shape flattening of the posterior membrane. Cross‐sectional area decreased to 0.41 cm^2^, an 85% reduction.

Dynamic imaging of the large airways during end‐inspiratory and forced expiratory maneuvers is widely used to evaluate the reduction of cross‐sectional airway lumen area, with a similar diagnostic criterion to bronchoscopy used (Figure 2) (Mitropoulos et al., 2021). Although one study found a high sensitivity of 97% for detecting airway malacia using this criterion, more conservative thresholds of collapse >70% have been suggested due to the healthy, asymptomatic individuals that also meet this criterion during forced maneuvers (Boiselle et al., 2009; Lee et al., 2007). Cine magnetic resonance imaging (MRI) offers an alternative technique to assess large airway dynamics without radiation exposure, allowing multiple respiratory maneuvers at different tracheal levels (Heussel et al., 2004; Williams et al., 2024) assessment method is not commonly used, but the studies also employed the 50% collapse diagnostic threshold.

### 5.2 Exercise assessment

Despite extensive literature assessing LAC during forced maneuvers at rest, very few studies have evaluated these conditions during real‐world exercise scenarios. Weinstein et al., (2016) were among the first to evaluate exercise‐associated EDAC in military personnel reporting exertional symptoms. Despite normal PFTs, bronchoprovocation testing, and CPET, dynamic CT imaging revealed EDAC in four subjects, while two others underwent exercise bronchoscopy on a cycle ergometer, which provided anatomical assessment at a time commensurate with symptoms.

**FIGURE 3:**
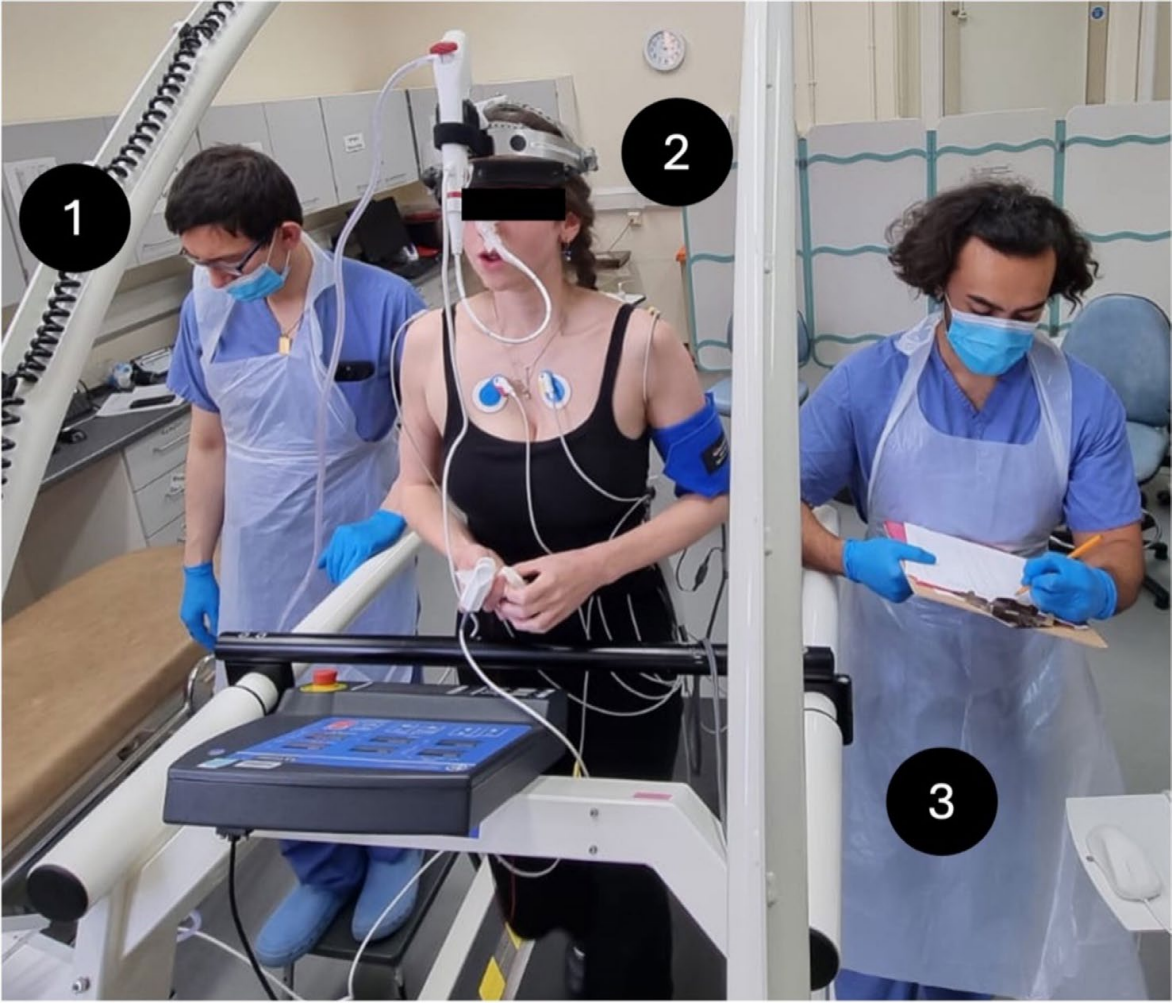
Continuous bronchoscopy during exercise (CBE) assessment of a 25‐year‐old female participant. (1) Experienced pulmonologist reviewing airway images. (2) Participant exercising with bronchoscope in‐situ. (3) Exercise physiologist recording vital sign measures and ratings of perceived exertion.

This approach was further developed by Williams et al., (2024) using CBE to visualize the large airway response during ambulatory, incremental exercise (Figure 3). In a group of healthy individuals, the assessment was feasible, did not require sedation, and permitted the characterization of the large airways during vigorous exercise. It also found the airway closure during forced expiration was markedly different from the response of the large airways at peak exercise). However, there were some methodological and safety considerations to be noted. While the cited study on healthy individuals reported no serious adverse events, dysrhythmia, or exercise‐induced syncope, common tolerability issues included sore throat/hoarse voice (40% of subjects) and transient voice changes (12%), with one case of mild, reversible bronchoconstriction following the procedure. Mild discomfort from topical anesthetic (64%) and bronchoscope placement (50%) was also reported. Furthermore, the importance of close cardiovascular monitoring during the CBE procedure is stressed, as there were two cases of transient desaturation and incidences where exercise was terminated due to elevated blood pressure (systolic pressure >225 mmHg). The precise causes for these abnormal findings are not yet clear, but these findings prompted the investigators to terminate exercise, and in all cases, the values rapidly returned to baseline. Regardless, these findings underscore the need for more studies before this is safely established in clinical practice.

The apparent disconnect between resting diagnostic findings and exercise‐related airway function may explain the inconsistent relationship between LAC severity and functional outcomes. Further research employing exercise‐based assessment techniques in symptomatic patients is needed to better understand the large airway response during physical activity and potentially provide new insights into unexplained exertional dyspnea.

## 6 TREATMENT APPROACHES

Currently, the treatment options available for LAC‐associated exertional symptoms are limited. Firstly, conservative approaches including optimizing management of comorbid conditions (i.e., treatments for COPD, asthma, gastro‐esophageal reflux disease, inducible laryngeal obstruction etc) may help discriminate LAC‐related symptoms (Swenson & Majid, 2022).

A current mainstay of treatment is physiotherapy techniques. We have advocated using an ‘ABC’ approach; this includes (A) airway clearance, (B) breathing pattern control and improve (C) capacity for exercise may reduce the burden of exertional symptoms and improve overall exercise tolerance (Grillo et al., 2022). The airway clearance arm of the model addresses the subjective and objective assessment of an effective cough. In the assessment of breathlessness; symptom severity, triggers and breathing pattern control are evaluated with the aim of optimizing these components. Finally, techniques to support improving exercise tolerance aim to optimize expiratory airflow and improve functional assessment measurements (i.e., 6MWT distance). For additional detail on the ABC model, please see Grillo et al., (2022).

With regard to LAC‐specific treatment options, the use of ambulatory continuous positive airway pressure (CPAP) may act as a “pneumatic stent” to maintain pressure in the airway permitting airway patency. Currently, this approach has been used anecdotally and been found to improve 6MWT distance and reduce respiratory neural drive (Kaltsakas et al., 2018). This is an area that requires further work in a formal, pragmatic setting to determine the impact of CPAP on exercise tolerance and physiological response.

Further steps can be taken in severe LAC groups reporting exertional symptoms such as mechanical airway stenting and tracheobronchoplasty (TBP). In severe cases of TBM and after an initial evaluation that includes assessment of PFTs, 6MWT and bronchoscopy to determine the degree and extent of airway collapse, a two‐week stent trial may be performed to stabilize the tracheobronchial tree. The criteria used to determine the effectiveness of the stent trial requires improvement in subjective respiratory quality of life/symptom burden and objective improvement in PFTs and/or 6MWT distance. Ernst et al. (Lazzaro et al., 2019) reported stent‐associated improvements in health‐related quality of life and FEV1 (but not 6MWT distance) in 58 participants undergoing stent trials; however, they also documented a high rate of stent‐related complications. Among the 58 patients, the main complications observed were partial stent obstruction (21 cases, 36.2%), infections (14 cases, 24.1%), and stent migration (10 cases, 17.2%), although these were generally reversible.

If short‐term stent placement is successful, TBP is a surgical option for severe cases. This procedure is often considered for patients with EDAC, where the cause of collapse is displacement of the posterior tracheal membrane, although the majority of the literature evaluates TBM patients. A polypropylene mesh is used to splint and stabilize the posterior tracheal and mainstem bronchial membranes during a TBP procedure. This option has been performed via both an open and a robotic surgical approach (Majid et al., 2008). In one series of 42 TBM patients who underwent TBP, the majority (82%) of patients reported overall satisfaction with the procedure, with a sustained improvement found in PFTs and a mean difference of 217 m in 6MWT distance (Ernst et al, 2007). However, post‐operative complications are common, and the evidence published is from specialist centres with experience in selecting appropriate candidates (Lazzaro et al., 2019). A prospective study reported surgical complications in 43% of patients. Common issues included prolonged mechanical ventilation (20%), pneumonia or bronchitis (11%), subcutaneous emphysema (11%), and atrial fibrillation (8%). The study also documented a 5.7% mortality rate, attributed to events such as pulmonary embolism and acute exacerbations of underlying lung disease (Majid et al., 2008).

## 7 FUTURE CONSIDERATIONS

Future research should aim to use airway visualization approaches during exercise to provide a real‐time assessment of airway dynamics at a time commensurate with symptoms and during physiologically relevant challenges (i.e., ambulatory exercise). These types of methods can assess clinical populations of interest to help determine the physiological limitation of LAC.

It is clear an improved understanding of the physiological mechanisms that underpin LAC‐associated symptoms is required. It may be of interest to assess the pressure changes in the large airways during heightened ventilatory challenges and exercise, which may help establish the cause of the patient's symptoms. An increased understanding of the mechanisms that cause trachealis muscle bowing and measuring associated changes in the work of breathing would also provide valuable pathophysiological insights. Other exercise‐related airway problems, such as exercise‐induced laryngeal obstruction, are associated with an increased respiratory neural drive and work of breathing, which is often suggested to underpin the cause of exertional dyspnea (Walsted et al., 2018). A similar mechanism may be occurring in patients with LAC. In addition, further evaluation of breathing pattern disorder involvement may provide insight into symptom development, as it is postulated that altered breathing mechanics might contribute to symptoms or compensate for airway instability.

Continued development and standardization of objective classification systems used to assess LAC, moving beyond the arbitrary 50% collapse threshold, is also needed.

Currently, the comparisons of novel approaches with standard clinical practice are limited due to the differences in methodologies (i.e., exercise compared to forced expiration). It may be useful to study the impact of airway collapse on exercise with imaging during exercise as well as novel bronchoscopy assessments.

Finally, it would be of interest to study if the CPAP‐induced splinting changes the airway appearance during exercise and correlates with reduced symptoms.

## 8 SUMMARY

To summarize, the large airway response to heightened scenarios of ventilation is complex. The precise definitions and prevalences of abnormal large airway function are yet to be established.

The majority of diagnostic assessments are performed at rest, yet there appears to be a discrepancy between the degree of collapse during forced expiratory maneuvers and what is evident during exercise. New emerging strategies using exercise assessments may provide valuable insight into this large airway response during real‐world practice. But further study is needed in clinical cohorts of interest.

## FUNDING INFORMATION

Giovanni Cenerini has received funding from the European Respiratory Society Clinical Training Fellowship 2024 for this work. Zander J. Williams salary is funded by the RELACS charity, part of Royal Brompton and Harefield Hospitals Charity.

## CONFLICT OF INTEREST STATEMENT

The authors have no perceived conflict of interest to declare.

## ETHICS STATEMENT

Ethical approval for this article was not required.

## PERMISSION TO REPRODUCE MATERIAL FROM OTHER SOURCES

Appropriate journal specific permission has been received to reproduce material from other sources. Please see figure legends where applicable.
